# Effective knowledge management in translational medicine

**DOI:** 10.1186/1479-5876-8-68

**Published:** 2010-07-19

**Authors:** Sándor Szalma, Venkata Koka, Tatiana Khasanova, Eric D Perakslis

**Affiliations:** 1 Centocor R&D, Inc. 3210 Merryfield Row, San Diego, CA 92130, USA; 2 GeneGo, 169 Saxony Road, #104, Encinitas, CA 92024, USA; 3 Centocor R&D, Inc. 145 King of Prussia Rd., Radnor, PA 19087, USA

## Abstract

**Background:**

The growing consensus that most valuable data source for biomedical discoveries is derived from human samples is clearly reflected in the growing number of translational medicine and translational sciences departments across pharma as well as academic and government supported initiatives such as Clinical and Translational Science Awards (CTSA) in the US and the Seventh Framework Programme (FP7) of EU with emphasis on translating research for human health.

**Methods:**

The pharmaceutical companies of Johnson and Johnson have established translational and biomarker departments and implemented an effective knowledge management framework including building a data warehouse and the associated data mining applications. The implemented resource is built from open source systems such as i2b2 and GenePattern.

**Results:**

The system has been deployed across multiple therapeutic areas within the pharmaceutical companies of Johnson and Johnsons and being used actively to integrate and mine internal and public data to support drug discovery and development decisions such as indication selection and trial design in a translational medicine setting. Our results show that the established system allows scientist to quickly re-validate hypotheses or generate new ones with the use of an intuitive graphical interface.

**Conclusions:**

The implemented resource can serve as the basis of precompetitive sharing and mining of studies involving samples from human subjects thus enhancing our understanding of human biology and pathophysiology and ultimately leading to more effective treatment of diseases which represent unmet medical needs.

## Background

The effective management of knowledge in translational research setting [1,2] is a major challenge and opportunity for pharmaceutical research and development companies. The wealth of data generated in experimental medicine studies and clinical trials can inform the quest for next generation drugs but only if all the data generated during those studies are appropriately collected, managed and shared. Some notable successes have been already achieved.

Merck has developed a system which enables sharing of human subject data in oncology trials with the Moffit Cancer Center and Research Institute [3]. This system is built from proprietary and commercial components such as Microsoft BizTalk business process server, Tibco and Biofortis LabMatrix application and does not address any data sharing issues outside of the two institutions.

There is a growing set of data being deposited in NCBI GEO [4], EBI Array Express [5], Stanford Microarray Database [6] and the caGRID infrastructure [7] which is derived from gene expression experiments on tissue samples collected from clinical setting. Many of those are from either drug discovery or biomarker discovery projects. In particular, Johnson & Johnson through its subsidiaries have contributed such data sets into GEO and Array Express.

These databases enforce standards for some of the elements of the experimental metadata [8]. In general, the phenotype annotations in the metadata are not required to follow standard dictionaries or vocabularies. That can cause considerable issues as it was recently demonstrated [9] and described in the following example. These databases allow bioinformaticians to download the normalized data and carry out further analysis. The typical setting for such analyses that the scientist poses some hypotheses with respect to the phenotype and the informatician then needs to discern those phenotypes from the semi-structured data and correlate it with genotype in a sub-optimal process. In some cases the decoding and interpretation of the different phenotype can lead to serious mistakes such as the case recently discovered when multiple publications interpreted normal samples as cancer samples leading to erroneous conclusions [9].

The computational experiments can lead to validation of the primary findings or to novel discoveries such as in the case of meta-analysis of multiple datasets. The burden of deconvoluting the phenotypes from source files downloaded from these primary sources and coding them in a standard to enable large-scale meta-analyses makes these types of discoveries very costly and in fact quite rare [10-13].

Data curation is a way to tackle some of these issues. Typically, derived databases of omics experiments are curated to create comparisons for specialized mining with specific questions in mind. For example, there are multiple resources being developed to integrate and analyze gene expression and other omic data and create contrasts (A vs. B comparisons) or signatures [14,15]. The limitation of these resources is that they strive to answer specific questions and thus limit in-depth exploration of the data.

The treasure trove of high-content data derived from human samples can be much more effectively mined if standard dictionaries applied to all these studies and each subject's clinical and the associated sample's genomics data is stored and analyzed through a system which enables efficient access and mining. An example of such standardized infrastructure and potential for pre-competitive sharing is presented below.

## Methods

Johnson & Johnson has invested in translational research by establishing within its pharmaceutical R&D division a set of translational and biomarker groups and focusing also on the management and mining of the data emanating from integrative settings crossing the drug discovery and development stages. One of the deliverables of this enhanced governance structure was the development of a translational medicine informatics infrastructure. This infrastructure is a combination of dedicated people, robust processes and informatics solution - tranSMART.

We have established a strong cooperation across the R&D of the pharmaceutical companies of Johnson & Johnson and an open innovation partnerships with the Cancer Institute of New Jersey and St. Jude Children's Research Hospital [16]. The R&D Informatics and IT group works in close collaboration with discovery biologists, pharmacologists, translational and biomarker scientist, clinicians and compound development team leaders with a goal to develop a system which enables democratic access to all the data generated during target validation, biomarker discovery, mechanism of action, preclinical and translational studies and clinical development.

An important aspect of successfully introducing a paradigm shift within a large pharmaceutical organization is change management. From the start we have recruited biologists, pharmacologists and physicians from various therapeutic areas to help champion the adaptation of the newly developed translational infrastructure but also to guide us through the development of the application in an agile environment.

**Figure 1:**
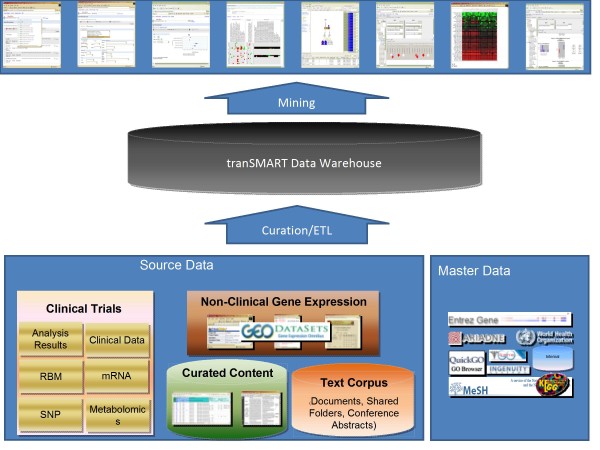
**Diagram of the tranSMART system**. Public and private data from multiple modalities (e.g.: gene expression, SNP, protein expression, etc) and areas (clinical and pre-clinical) are aligned to standard ontologies and curated and undergo ETL processing to be stored in a central data warehouse. A variety of user interfaces are implemented based on open-source components to enable data query, analysis and mining.

The translational medicine data warehouse - tranSMART - was developed in partnership with Recombinant Data Corporation (Fig. 1) and detailed description of the system was reported previously [17]. Here we give an overview of the salient points of the application. In short, the data warehouse contains structured data from internal clinical trials and experimental medical studies and a set of public sources. The data modalities include clinical data and aligned high-content biomarker data such as gene expression profiles, genotypes, serum protein panels, metabolomics and proteomics data.

Data is stored in an Oracle 11 database which is fully auditable. We selected a set of open-source components to assemble the application in contrast to the strategy followed by Merck. A user interface providing a biological concept search of analyzed data sets and an i2b2 [18] based comparison engine for subject level clinical data were constructed in Java using GRAILS. Advanced pipelines were established for launching analytical workflows of gene and protein expression and SNP data between cohorts to present comparisons in Gene Pattern [19] and Haploview [20]. The solution is hosted on Amazon Elastic Compute Cloud and strict security policy is enforced. Authentication as well as role-based authorization model is implemented throughout the application so that study level permissions are enabled.

Clinical trial and experimental medicine study data sets were transformed by curators and ETL (Extract, Transform, Load) developers into an i2b2 [21] EAV (Entity-Attribute-Value model) structure and a standardized ontology based on CDISC SDTM (Clinical Data Interchange Standards Consortium - Study Data Tabulation Model) [22] was applied. Currently, the system contains a growing number of curated internal studies - at the time of writing it is 30 across immunology, oncology, cardiovascular and CNS therapeutics areas.

**Figure 2:**
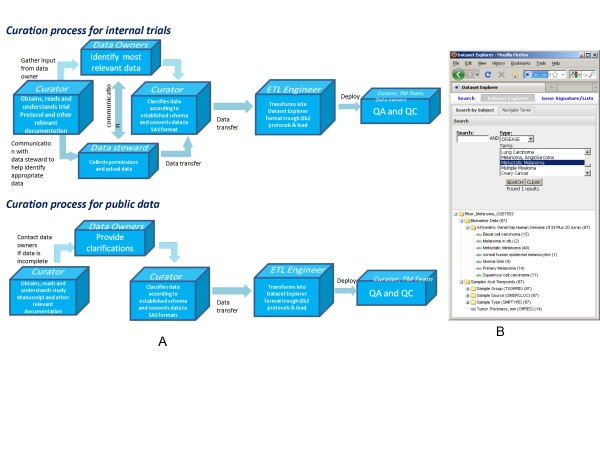
**Curation process**. Curation process diagram describes data flow for both public and internal data. (a). Public study (GSE7553) from NCBI GEO was curated and uploaded into tranSMART. CDISC SDTM codes are applied for concepts such as Tumor Thickness - ORRES and standardized concepts help the user navigate through complex studies (b).

The same process was utilized for multiple public expression experiment from samples of human origin downloaded from GEO, Array Express or other public repositories (see the flow chart and example in Figure 2a, b). The gene expression data was normalized using a standard protocol if the original raw files were available or the intensities were downloaded from the source systems. The phenotypes were manually turned into CDISC SDTM concepts which then were stored in a standardized hierarchy accessible through the familiar explorer paradigm. Here each concept can be selected and used for constructing a query. At the time of writing this article there are 30 such data sets in tranSMART.

## Results

**Figure 3:**
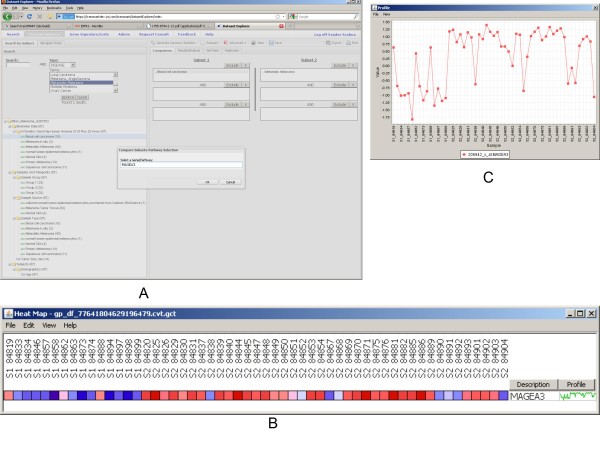
**Hypothesis re-validation**. Original findings can be re-validated by using a simple drag-and-drop cohort selection and analysis paradigm such as visualizing *MAGEA3 *differential expression between basal cell carcinoma and metastatic carcinoma samples (a-c).

In the following we show some sample analyses which can be done very efficiently with the tranSMART system once appropriate curation of public data [23] takes place (Fig. 3a-j). With a simple drag-and-drop cohort selection paradigm different dimensions of the data can be selected and the system can run queries in mere seconds to generate analyses which can reproduce original results such as *MAGEA3 *differential expression between basal cell carcinoma and metastatic carcinoma samples shown in Figures 3a-c.

**Figure 4:**
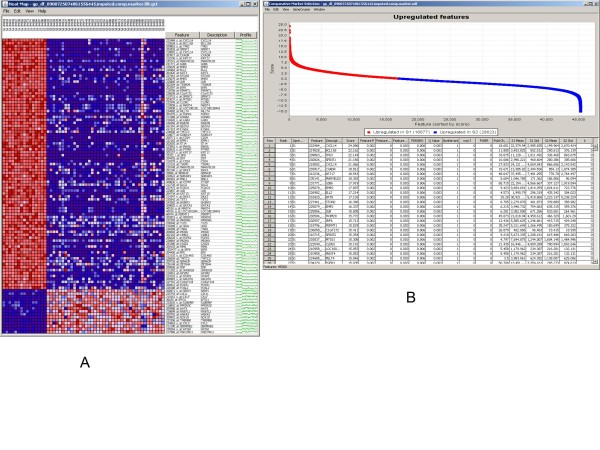
**ComparativeMarkerSelection**. Original analyses can be redone using a different methodology such as comparing basal cell carcinoma samples with metastatic carcinoma samples using the ComparativeMarkerSelection algorithm (a,b).

**Figure 5:**
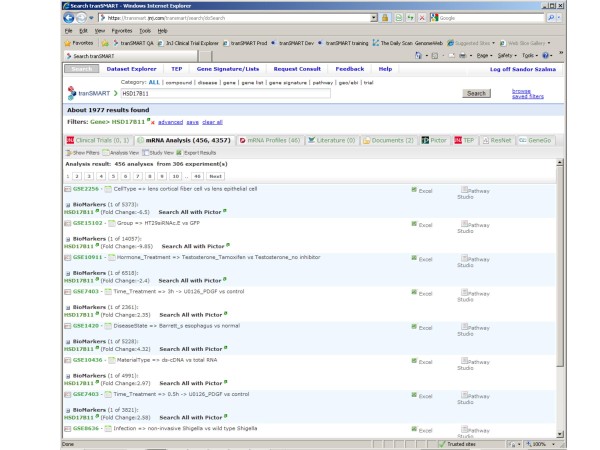
**Search**. Searches can be run for discovering the associations of concepts found in analysis across multiple databases.

Interestingly, comparing basal cell carcinoma samples with metastatic carcinoma samples using the ComparativeMarkerSelection algorithm [24] built into GenePattern highlights the *HSD17B11*gene as the highest scored gene which is consistently upregulated in the metastatic samples (Figure 4d, e) supported by the sophisticated statistical algorithms built into the GenePattern application (e.g.: false discovery rate estimation by the Benjamini and Hochberg procedure [25]). Searching for evidence in PubMed for association between *HSD17B11 *and melanoma brings up no hits but is associated with seminal vesicle invasion in prostate cancer [26]. TranSMART system also enables doing a thorough search across multiple databases for evidence of a gene's involvement in biological processes and experiments as illustrated in Figure 5.

**Figure 6:**
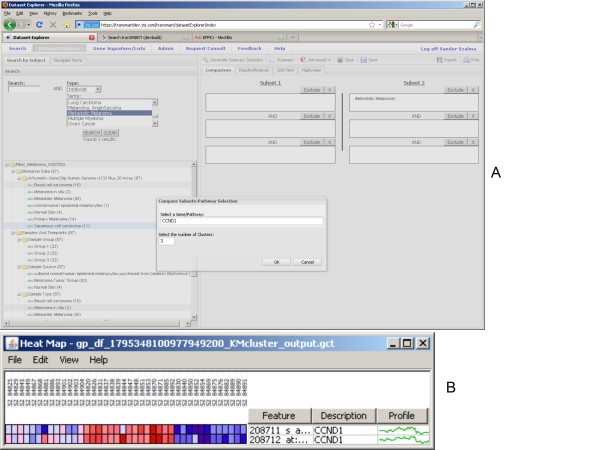
**New hypothesis testing**. New hypotheses can be tested - the role of *cyclin D1 *in metastatic melanoma in single cohort using k-means clustering (a,b).

Novel hypotheses can be also tested in a straightforward manner as it is illustrated in Figures 6a, b. Here the suggested association of *cyclin D1 *with progression from benign to malignant stages [27] is illustrated using k-means clustering as one of the clustering methods implemented through connection with GenePattern [19]. While the expression levels of *cyclin D1 *increase from benign to malignant, in metastatic melanomas the expression level decreases [27] which in turn demonstrated by the clustering method clearly delineating multiple subgroups of samples in the presumably homogenous metastatic melanoma cohort.

**Figure 7:**
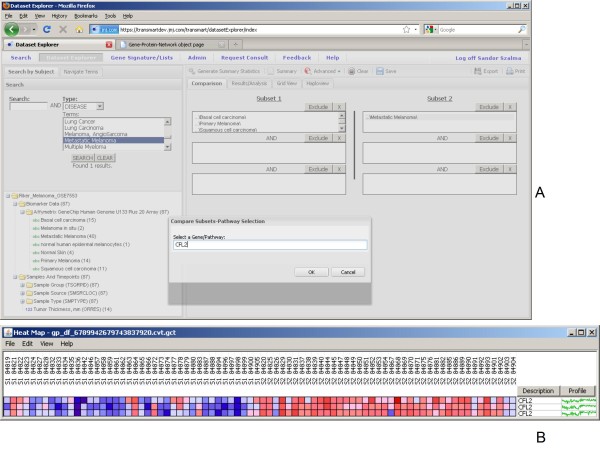
**Combined analysis**. New analyses can be run - e.g.: contrasting combined primary melanoma, basal and squamous cell carcinoma vs. metastatic melanoma (a,b).

Queries can use Boolean operators such as OR and AND as illustrated in Figures 7a, b where the first cohort contains samples from tissues from subjects with primary melanoma, or basal cell carcinoma or squamous cell carcinoma and the second cohort consist of samples from tissues from subject with metastatic melanoma. The example shows the resulting heatmap of expression data of a particular gene (*CFL2*) of this complex query. In subset one (denoted by S1_... sample ids) most of the samples have low expression of the gene of interest (denoted by blue color) whereas in subset two (denoted by S2_... sample ids) most of the samples have high expression of the gene of interest (denoted by red color).

**Figure 8:**
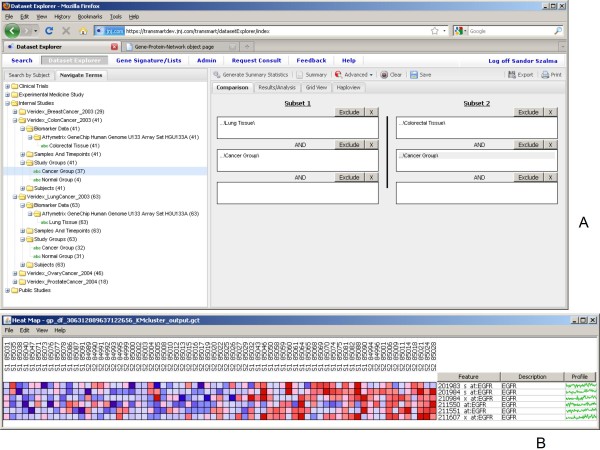
**Meta-analysis**. Comparing lung cancer and colorectal cancer gene expression data from multiple experiments using k-means clustering with the *EGFR *gene where k = 2.

Cross-study meta-analyses are also available in the application (Figure 8a, b). In this example two gene expression datasets from Veridex - from colorectal and lung cancer tissue samples [9] - were previously processed, normalized and uploaded into tranSMART. Both sets of tissues were analyzed using the same Affymetrix U133 GeneChip platform [9]. The tranSMART system then enables one to construct a query where the gene expression values of the two sets of tissue samples can be aligned and analyzed. As an example we show that a simple k-means clustering as implemented in GenePattern using the *EGFR *gene with k = 2 can stratify the subjects into high and low expressors.

## Discussion

The tranSMART system allows clinicians, translational scientists and discovery biologists to interrogate aligned phenotype/genotype data to enable better clinical trial design or to stratify disease into molecular subtypes with great efficiency. Initial successes of applying this system point towards the high value of translational data in proposing indications for drugs with new mechanism of actions [J. Smart, personal communication] and selecting biomarkers for stratified medicine.

The system has been in wide use across multiple therapeutic areas within the pharmaceutical companies of Johnson and Johnson. Comparing biological processes and pathways between multiple data sets from related disease or even across multiple therapeutic areas is an important benefit of such a system. Through the examples presented above we have shown that the tranSMART system allows scientist to quickly re-validate hypotheses or generate new ones with the use of an intuitive graphical user interface. The use cases supported by tranSMART have been developed in close collaboration with key users and the solution was built from many open source systems making the adaptation of the system straightforward.

We have implemented a fine-grained, role-based authorization model throughout the application so that study level permissions are enabled and can be controlled by the study owners. During curation the study owners are actively involved in reviewing and approving the loading and standardization of the data from their studies. This approach greatly enhanced the cooperation of the study owners and the ultimate success of the data warehouse.

## Conclusions

A well-constructed system can enable scientist to test but also generate new hypotheses using well-curated, high-content translational medicine data leading to deeper understanding of various biological processes and eventually helping to develop better treatment options.

Active curation and enterprise data governance have proven to be critical aspects of success. The capability of the system to query both internal and public data and that during the development and implementation full organizational alignment was ensured turned out to be crucial.

Because large part of tranSMART is built from open source systems it is much more amenable for being shared with academic institutions in a pre-competitive setting enabling collaborations aimed at developing deeper understanding disease biology.

The tranSMART system is under active development including active curation of additional studies, implementing new modalities and adding novel workflows. Future development may include connection to the internal biobank. By the established system and the robust processes our research and development organization can now effectively manage not just the complex and multimodal data but can also unlock the potential of the data by transforming it into actionable knowledge.

## Competing interests

SS, VK and EP are employees of Johnson and Johnson.

## Authors' contributions

SS and EP conceived and designed the study. VK and TK assisted with the experiments. SS drafted the manuscript. All authors read and proofed the final manuscript.
